# Bio-inspired pores for selective ion transport

**DOI:** 10.1093/nsr/nwaf025

**Published:** 2025-01-23

**Authors:** Ben Corry, Ruitao Jin

**Affiliations:** Research School of Biology, Australian National University, Australia; Research School of Biology, Australian National University, Australia

The ability to selectively transport specific ions across membranes is critical in biology, underlying processes such as the transmission of nerve impulses, stimulation of muscle contraction and the regulation of cell volume and blood pressure [1]. Copying this selectivity in synthetic materials has great potential in applications such as energy conversion, sensing and water filtration [2]. However, achievement of the degree of selectivity that is seen in biology in synthetic materials remains challenging, especially for the highly similar Na^+^ and K^+^ ions.

**Figure 1.**
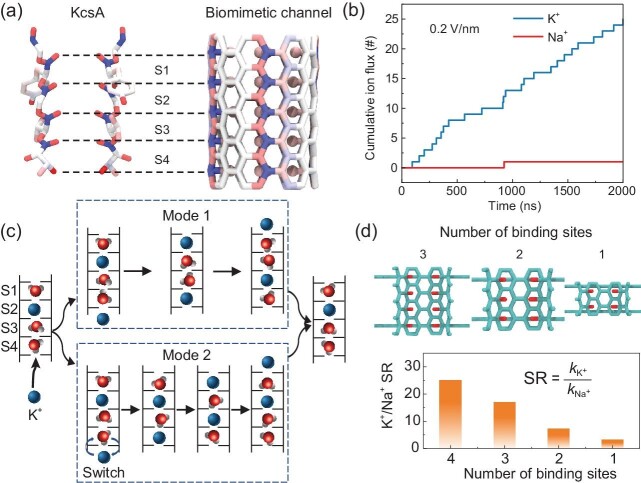
(a) A biomimetic pore is constructed based upon the structure of the KcsA potassium channel. (b) Molecular dynamics simulations under an applied field show selective conduction of K^+^ over Na^+^. (c) Ion permeation takes place in a knock-on mechanism with water molecules between adjacent ions. (d) The selectivity scales with the number of ion binding sites. Adapted with permission from [3].

In their recent study, Zhu *et al*. [3] followed the preceding approach of learning from the geometry and chemistry that are found in biological channels within a carbon nanostructure to see whether similar degrees of selectivity could be achieved [4–7]. To do this, they first constructed a stretched carbon nanotube (CNT) model and then layers of carbonyl oxygen atoms were introduced into the pore interior to mimic the spatial arrangement of oxygen atoms within the selectivity filter of KcsA along the main axis (Fig. 1a). They found that this pore has significant ion selectivity for K^+^ over Na^+^ in MD simulations (Fig. 1b). As in biological ion channels, knock-on conduction is observed (Fig. 1c). In this, water molecules and K^+^ ions can occupy the binding sites within the CNT and the confined space in the channel forces both K^+^ and Na^+^ to lose most of their hydrating water molecules. Meanwhile, the inwardly facing carbonyl oxygen atoms compensate for the loss of the hydration shell, as occurs in the selectivity filter of potassium channels. The number of ion binding sites in the biomimetic channel negatively correlates with ion selectivity (Fig. 1d), as fewer and lower energy barriers reduce K^+^ sensitivity but increase ion permeation efficiency.

Despite copying the geometry of biological pores, there are a number of differences between the ion transport seen in the functionalized CNTs in this study and their biological counterparts. Here, the permeating K^+^ ions are most commonly separated by two water molecules. In contrast, debate continues as to the exact configurations in the biological context. Ions are found to have only one [8] or no water molecules [9–11] between them. Although this sounds like a minor difference, some argue that direct contact of K^+^ is essential to achieve high flux and selectivity [10]. In the current study, Na^+^ ions are also seen to pass close to the pore walls between two adjacent carbonyls, which is more similar to the biological case in which Na^+^ passes centrally in plane with the carbonyls [12] but can move off axis between them [13]. One difference is that biological proteins are flexible and can relax to snugly coordinate with a centrally placed Na^+^. In contrast, the CNT is rigid and the locations of the carbonyl groups are relatively fixed. This could enable the geometry to better coordinate passing K^+^ than Na^+^ according to the ‘snug fit’ model as was originally proposed but later disproved for the biological channels [14].

The demonstration of selectivity in simulations is a significant achievement and can aid in understanding the different physical mechanisms that can be utilized to achieve selective transport. One remaining challenge is the difficulty in manufacturing the precise geometries of the pores described by Zhu *et al.* The proposed pore requires careful placement of the carbonyl groups within the CNT interior, including challenging chemistry to attach the functional groups, and the pore had to be stretched. Such precise arrangements are difficult to control and selectivity may not be retained if the carbonyl groups are placed in different configurations in the pore or on its exterior. Adding them may also require the introduction of additional defects to the carbon lattice that are not captured here, which could influence transport. There have, however, been significant advances in the controlled manufacture of nanostructured pores, which means that the manufacture of selective pores such as those described by Zhu *et al.* may be achieved in the future. Methods are being developed to reduce the size distribution of CNTs [15,16], as a synthesized array will have to include a range of lengths and widths. Various groups have managed to functionalize the inner walls of nanotubes by oxidizing via HNO_3_ treatment [17], thermal annealing [18] or plasma functionalization [19]. There is also evidence that CNTs can be stretched significantly without breakage [20]. These studies do, however, highlight a significant difference between the biological and synthetic systems. Although biological pores are large and complex molecules, they are reproducibly produced by the cellular machinery so that every pore is chemically identical. In contrast, synthetic porous materials contain heterogeneity in size, shape and chemistry that will need to be controlled to generate precise functions.

In conclusion, Zhu and co-workers propose a new model biomimetic channel that can achieve high ion selectivity like its biological counterpart that differs in geometry from previous examples [4–7]. Using MD simulations, they provide an explanation of how this selectivity is achieved. Although challenges remain to precisely manufacture these materials, significant progress is being made both computationally and experimentally to design new functional materials that can achieve a high level of ion selectivity for use in a variety of industrial applications.
